# Floating raft culture of *Gracilariopsis* *longissima* for optimum biomass yield performance on the coast of Cox’s Bazar, Bangladesh

**DOI:** 10.1038/s41598-023-28675-0

**Published:** 2023-02-09

**Authors:** Mohammad Khairul Alam Sobuj, Md. Golam Mostofa, Zahidul Islam, Ahmad Fazley Rabby, Turabur Rahman, Saima Sultana Sonia, Shanur Jahedul Hasan, Shafiqur Rahman

**Affiliations:** Marine Fisheries and Technology Station, Bangladesh Fisheries Research Institute, Cox’s Bazar, 4700 Bangladesh

**Keywords:** Plant sciences, Ocean sciences

## Abstract

Seaweed cultivation is an eco-friendly method and attracts growing interest which needs a multi-criteria approach for its sustainability. In our present study, an economically significant red alga, *Gracilariopsis* *longissima* was cultured using a floating raft method on the coast of Cox’s Bazar, Bangladesh for a period of 90 days from January to March 2022. The effects of different factors such as rope materials, culture type, raft shape, seeding intensity, harvesting phase and water depth on the biomass yield production of *G.* *longissima* were evaluated during a 90-day culture period. The biomass yield production and daily growth rate (DGR% day^−1^) were estimated to evaluate the possibilities of *G.* *longissima* cultures in a floating raft culture method. The range of biomass yield production (3.03–13.37 kg/m^2^) and DGR (3.08–4.72% day^−1^) is satisfactory in the floating raft culture method. Different water quality variables, the seasonal appearance of epiphytic algae and a cost–benefit analysis of seaweed culture were also performed. A total of eight epiphytic algal species were recorded, which resulted in major challenges for the growth of *Gracilariopsis*. The per month income for a seaweed farmer was estimated to be US$175.17 for 20 rafts. Our research concluded that farming of *G.* *longissima* in the floating raft method could be successfully performed from January to March on the coast of Cox’s Bazar.

## Introduction

Globally, the significant application of seaweed is found in food, animal feed, cosmetics, phycocolloid or hydrocolloid industry, pharmaceutical industry, biofuel production, fertiliser, wastewater treatment and bioremediation^1^. Additionally, this marine flora is a source of diverse bioactive compounds such as saponin, terpenoid, phenol, flavonoid and prominent natural antioxidant compounds^2,3^. Their versatile application has made this category extremely popular in the last decade. The alga *Gracilariopsis* *longissima* (Rhodophyta) belongs to a family of red macroalgae which has been widely authenticated by their value as agarophytes. This seaweed contains agar (gelling carbohydrates) in their cell walls and intercellular matrices that is used in the hydrocolloid industry^4^. Approximately 100 species of red algae, including *Gracilariopsis* spp., are cultured worldwide for the agar industry. Seaweed cultivation is considered to be a clean and environmentally friendly income generation method and provides important ecological services in coastal waters^5^. Seaweed has been cultivated for centuries in Japan, China, Korea and other Asian countries though culturing seaweed is still in its early stages in Bangladesh. The world aquaculture production of marine macroalgae has increased dramatically, up from 10.6 MT in 2000 to 32.4 MT in 2018 while *Gracilaria* seaweeds (*Gracilaria* spp.) production has increased from 0.056 MT in 2000 to 3.45 MT in 2018^6^. This plant is regarded as a viable candidate for culturing in many parts of the world because of its geographical range and prompt growth rates^7^. This species has also been cultured worldwide in the open coastal waters of bays^8^, in Integrated Multi-Trophic Aquaculture (IMTA) systems^9^ and in integrated polyculture practices as biofilters^10^.

In the case of Bangladesh, seaweed farms are gradually springing up as a result of increased demand for raw seaweed and its derivatives, as well as the necessity for fishing communities to seek alternative or additional income. Consequently, wild raw materials are not always adequate to sustain a successful entity in the face of rising raw seaweed demand. Therefore, it is important for seaweed producers to be able to cultivate seaweed to keep up with rising requirements. Seaweed cultivation will also reduce pressure on natural stocks and help to have a continuous supply of raw material all year round. Furthermore, unlike other types of mariculture, like shellfish or fish farming, seaweed cultivation requires less investment and may even be expanded in conjunction with such cultures, enhancing profitability without the need for new complex infrastructure^11^. To execute culture on a commercial scale, it is required to create a simple and easy cultivation system with a suitable cost–benefit ratio, as well as an understanding of the physicochemical parameters of water, production and biomass yield for economically important species.

The final product quality largely depends on seaweed type, geographical location, water quality characteristics of the cultivation area, cultivation methods and processing conditions^12^. For the development of the cultivation technique of *Gracilaria* spp., several methods have been attempted on numerous occasions throughout the world, such as rectangular frame^11^, square frame^13^, long line and net method^14^ and rope hanging method^15^. In India, the raft method was successfully applied for the cultivation of several *Gracilaria* spp^16^. There have been a limited number of experiments on seaweed cultivation in Bangladesh with all of them focusing on optimising different factors and the adoption of different culture methods, depending on ecological characteristics at possible sites^17–19^. All of these experiments were only conducted between the intertidal zones and applied semi-floating single-line, square net or long line methods for seaweed cultivation. However, the main drawbacks of these approaches were crop loss owing to crops breaking from the base, especially during bad weather. No studies have been conducted on the possibility of using a floating raft culture beyond the intertidal zone in Bangladesh. As the demand for seaweed gradually increases in the domestic market, it needs an extension of seaweed culture beyond the intertidal zone by adopting some new culture methods. Additionally, there are always some aspects that influence seaweed production profitably to a significant extent. Therefore, the purpose of this study was to optimise the influence of various critical parameters like rope materials, culture type, raft shape, seeding intensity, harvesting phase and water depth on the biomass yield of seaweed for a floating raft culture method to investigate the possibility of *G.* *longissima* production in the Cox’s Bazar region. Furthermore, the cost–benefit analysis and presence of epiphytes during the seaweed culture period were also evaluated.

## Results

### Water quality variables

The cultivation of seaweed requires appropriate physicochemical conditions. The range values of different water quality variables during the 90-day culture period of Chowfoldondi in Cox’s Bazar are described in Table 1.

**Table 1:** Different water quality variables of the culture site during the experimental period (according to the references 11, 12, 19, 20 and 21).

No.	Parameters	Range	References				
			Bezerr and Marinho-Soriano^11^	Bokhtiar et al.^19^	Rejeki et al.^12^	Lee et al.^20^	Veeragurunathan et al.^21^
01	Temperature (℃)	24.1–32	27–30	21.9–22.1	20–34	30.3–31.1	26–31.9
02	pH	7.1–7.6	7.1–8.3	7.9–8.1	7–8	7.6–8.04	8.1–8.3
03	DO (mg/L)	6.11–7.93	–	7.1–7.3	–	5.89–7.45	4.51–7.32
04	Salinity (ppt)	30–36	30–36	31.7–32.3	5–35	32.9–35.0	30–35
05	Alkalinity (mg/L)	125–145	–	–	–	–	–
06	Ammonia (mg/L)	0.27–0.61	–	–	–	< 5	–
07	Nitrite (mg/L)	0.02–0.07	–	0.33–0.55	–	0.01–1.90	–
08	Nitrate (mg/L)	0.14–0.34	–	0.43–0.83	0.0–1.45	< 0.07	–
09	Phosphate (mg/L)	0.07–0.11	–	–	–	< 0.03	–
10	TDS (mg/L)	13.21–18.31	–	–	–	–	–
11	Silica (mg/L)	0.36–0.51	–	–	–	–	–
12	Transparency (cm)	40–50	30.2	73.1–75.9	21	–	–

### Effect of rope material

**Figure 1:**
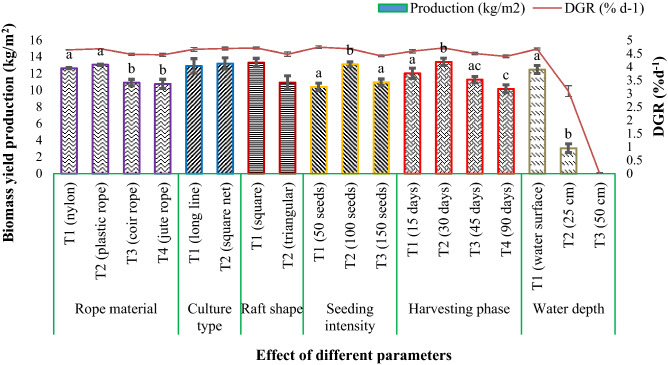
Effect of different parameters on the biomass yield production (kg/m^2^) and DGR (% day^−1^) of *G.* *longissima* in the floating raft method.

In this regard, we used four different treatments, where T_1_ = nylon rope, T_2_ = plastic rope, T_3_ = coir rope and T_4_ = jute rope. In this study, we observed that the plastic rope biomass yielded a higher production than the other three treatments. In the case of the nylon rope and plastic rope, we found that the plastic rope (13.02 ± 0.08 kg/m^2^, 4.66 ± 0.01% day^−1^) is yielded more biomass than the nylon rope (12.60 ± 0.06 kg/m^2^, 4.62 ± 0.01% day^−1^); whereas the coir rope and jute rope biomass yielded insignificant amounts in comparison. After all, the coir rope and jute rope showed significantly (p < 0.05, F value = 35.91) lower production than the nylon and plastic ropes (Fig. 1 and Table 2).

**Table 2:** One-way ANOVA and T-test results showing the effects of different parameters on the biomass yield production (kg/m^2^) and daily growth rate (% day^−1^) of *G.* *longissima* in the floating raft culture method.

Source of variation	d.f	M.S	S.S	F	P value
Rope materials					
Biomass yield production (kg/m^2^)	11	4.18	12.54	35.91	0.00
DGR (% day^−1^)	11	0.108	0.036	30.521	0.00
Culture type					
Biomass yield production (kg/m^2^)	5			0.243	0.648
DGR (% day^−1^)	5			0.269	0.631
Raft shape					
Biomass yield production (kg/m^2^)	5			1.135	0.347
DGR (% day^−1^)	5			1.910	0.239
Seedling intensity					
Biomass yield production (kg/m^2^)	8	6.919	13.838	45.053	0.00
DGR (% day^−1^)	8	0.171	0.085	62.478	0.00
Harvesting phase					
Biomass yield production (kg/m^2^)	11	5.560	16.680	23.564	0.00
DGR (% day^−1^)	11	0.049	0.146	22.953	0.00
Water depth					
Biomass yield production (kg/m^2^)	8	136.436	272.871	807.722	0.00
DGR (% day^−1^)	8	16.76	33.53	1343.94	0.00

*d.f.* degrees of freedom, *M.S.* mean squares, *S.S.* sum of squares.

### Effect of culture type

Here, we performed two treatments, T_1_ (long line) and T_2_ (square net), to discern their impact on the production of *G.* *longissima.* In the case of square net, we found a distinctively higher production and percent of daily growth rate than found on long line. The production and percent of the daily growth rate of long line and square net are 12.87 ± 0.87 kg/m^2^, 4.64 ± 0.07% day^−1^ and 13.17 ± 0.67 kg/m^2^, 4.67 ± 0.06% day^−1^, respectively. The outcome of the two methods is shown in Fig. 1 and Table 2.

### Effect of raft shape

In this case, we used two different raft shapes, T_1_ (triangular) and T_2_ (rectangular), to assess the effect of biomass yield performance on the raft shape of our cultivated species. It was found that a rectangular-shaped raft was more productive than a triangular-shaped raft (Fig. 1 and Table 2).

### Effect of seeding intensity

In this experiment, we used seeding intensity as a variable. Three treatments were tested in this experiment with the seeding intensities of 50, 100 and 150 seeds/m^2^. It was found that T_2_ (100 seeds/m^2^) showed a significantly (p < 0.05, F value = 45.053) higher production (13.06 ± 0.29 kg/m^2^) and reasonable percent daily growth rate (4.66 ± 0.02% day^−1^) (p < 0.05, F value = 62.478) than the other two treatments (T_1_ = production 13.06 ± 0.29 kg/m^2^ and % DGR = 4.72 ± 0.05% day^−1^; T_2_ = production 13.06 ± 0.29 kg/m^2^ and % DGR = 4.72 ± 0.05% day^−1^) (Fig. 1 and Table 2).

### Effect of harvesting phase

Four treatments were used (T_1_ = 15 days, T_2_ = 30 days, T_3_ = 45 days and T_4_ = 90 days) to evaluate the production performance of *G.* *longissima* depending on different harvesting phases. The study showed an increase in production and a percent daily growth rate in T_1_ and T_2_; whereas, they declined in T_3_ and T_4_. The result indicated that the cultured seaweed harvested at a 30-day interval showed higher (p < 0.05, F value = 23.564) production compared to other treatments (Fig. 1 and Table 2).

### Effect of water depth

To investigate the effect of depth on the production of seaweed, three treatments (T_1_ = water surface, T_2_ = 25 cm depth and T_3_ = 50 cm depth) were studied at our experimental site. Production in terms of depth in our study greatly varied. The results showed that the growth performance of *G.* *longissima* on the water surface is more highly productive (p < 0.05, F = 807.722) than at 25 cm and 50 cm depth. Treatment 2 resulted in very lower production during the study period; whereas, treatment 3 resulted in no production at all, as the seedlings died after a few days of their plantation (Fig. 1 and Table 2).

### Seasonal appearance of epiphytic algae

A total of eight algal species were recorded as epiphytes in the *G.* *longissima* culture (Table 3). Among them, six species were Chlorophyta, and two species were Rhodophyta. *Ulva* *intestinalis,* *Ulva* *compressa,* and *Ulva* *reticulate* were found in all three months of the culture period. The dominance of green epiphytic algae was observed throughout the entire culture period. The highest number of epiphytic algae (eight) was identified in March, while the lowest number (three) was documented in January.

**Table 3:** Monthly occurrence of epiphytic algae during the culture period.

Epiphytic algae	Months (2022)		
	January	February	March
*Ulva* *intestinalis*	+	+	+
*Ulva* *compressa*	+	+	+
*Ulva* *reticulata*	+	+	+
*Cladophora* *fascicularis*	-	+	+
*Chetomorpha* sp.	-	-	+
*Acanthophora* *spicifera*	-	-	+
*Polysiphonia* sp.	-	-	+
Total	3	4	8

“ + ” presence, “ − ” absence.

### Cost–benefit analysis of the seaweed culture

The biomass yield production from 20 rafts was 420 kg dry weight at the end of the second cycle, with a market value of US$1,688.40 (Table 4). Fresh biomass yield production was calculated after subtracting the seed materials. The cost–benefit analysis showed a US$525.5 profit for a 90-day culture period (one cycle) with 20 rafts. Thus, an individual seaweed farmer could make a profit of about US$175.17 per month through seaweed cultivation. We conducted a 90-day culture period of seaweed and calculated the possible economic return of a 180-day culture period. Four times the profit can be earned for a 90-day extension of the culture period as long as the input materials are the same.

**Table 4:** Estimation of the cost–benefit relationship of the *G.* *longissima* culture in a floating raft for a 180-day culture period.

	1st cycle			2nd cycle			Total
	1st harvest	2nd harvest	3rd harvest	1st harvest	2nd harvest	3rd harvest	
(A) Investment (US$ for 20 rafts/seaweed farmer)							
Input materials cost^a^ (bamboo, plastic drams, rope, protective net, anchors)	US$ 540.23			−			US$ 550
Maintenance cost	US$45.97			US$51.20			US$ 97.17
Total investment	US$586.20			US$51.20			US$ 637.4
(B) Biomass yield production							
Biomass fresh weight (kg/m^2^/harvest)	4.34	4.34	4.34	4.34	4.34	4.34	26.04
Biomass dry weight (kg/m^2^/harvest)	0.70	0.70	0.70	0.70	0.70	0.70	4.2
Biomass dry weight (kg/raft/harvest)	3.5	3.5	3.5	3.5	3.5	3.5	21
Biomass production (kg/20 raft/harvest)	70	70	70	70	70	70	420
Market value (US$4.02/kg)	281.4	281.4	281.4	281.4	281.4	281.4	1,688.40
Total income							US$1688.40
Total profit from biomass yield production/two cycle (B − A)							US$1051.00
Total profit from biomass yield production/cycle							US$525.5
Total profit from biomass yield production/month/seaweed farmer							US$175.17

^a^Exchange rate 1 US$ = 87.0 Bangladeshi Taka (BDT).

## Discussion

The method of seaweed cultivation always has a significant impact on both the biomass yield and the daily growth rate of seaweed^22^. In our present study the biomass yield performance of *Gracilariopsis* *longissima* was observed in a floating raft method. The cultivation of seaweed on a commercial scale was accomplished using the floating raft culture technique with great success in the case of *Gracilaria* *edulis*^22,23^, *G.* *verrucosa*^24^ and *G.* *debilis*^16^. The use of floating rafts in seaweed cultivation offers various assistances in comparison to other approaches. Seaweeds are grown at the surface of the water, where they receive increased sunlight exposure. The floating technique accelerates the gaseous exchange among the seaweeds and water by minimising sedimentation and self-shading problem. The use of a protective net at the lower portion of the bamboo raft reduces the biomass loss especially during adverse weather. The floating structure can easily raise and fall vertically during tidal action. Moreover, this structure makes it easier to manage and harvest of seaweed with easy reallocation if necessary.

It is well known that growth of seaweed is largely influenced by water quality variables^11,21^. In our experiment, different water quality variables showed lower fluctuations throughout the culture period due to low rainfall and surface runoff. The water temperature fluctuated from 24.1 to 32 °C, which is within the recommended temperature range (26–31 °C) for *G.* *dura* cultures^21^. It was found that the biomass yield production was negatively correlated with the seawater temperature as excessive exposure to sunlight and temperature could result in physiological disruption of the seaweed^10,21^. Salinity is a crucial factor for *Gracilariopsis*’s culture as the growth of *Gracilariopsis* is largely limited by the adequacy of salinity, despite the presence of other nutrients such as phosphate^12^. During our experiment, the salinity in the culture area was between 30 and 36 ppt (Table 1). It was found that *G.* *longissima* can adapt to a wide range of salinities (17–38 ppt) to respond to abiotic alterations^25^. When the salinity range was between 30 and 35 ppt, the highest daily growth rate was observed, and when the salinity level went down below 15 ppt, a partial or total tip discolouration was observed in the case of *G.* *longissima*^25^*.* Nitrate improved the biological activity of several *Gracilaria* species^22^. The levels of nitrate (mg/L) and nitrite (mg/L) were found to be at a satisfactory level throughout the experimental period. The Secchi disc transparency level at the culture site was about 40–50 cm, and the average water depth of the culture site was about 700 cm. *G.* *gracilis* can be cultured up to 400 cm with a higher Secchi depth (450 cm) as described by Mensi et al.^26^. A low level of transparency in our culture sites could have been caused by an increase in turbidity as a result of a muddy bottom, wind action or navigation.

Concerning the effect of rope materials, plastic rope showed the highest biomass yield performance and %DGR followed by nylon, coir and jute rope. Veeragurunathan et al.^21^ also observed maximum biomass yield in *G.* *dura* cultures by the polypropylene net method and Kaliaperumal et al.^14^ observed lower growth rate in *G.* *edulis* cultures by the coir rope method. Coir and jute ropes are biodegradable and can not sustain long periods in saline water. These types of ropes are environmentally friendly but are not sustainable for longer than two months in the culture system. The use of coir and jute ropes in the seaweed culture caused extra maintenance costs. But, if the raw materials are the farmer’s own materials, it will minimise the input materials’ cost. Apart from specific and geographical differences, it was revealed that cultivating method has a significant impact on red algae biomass yield and the daily growth rate^22^. In our present investigation, we found distinctively higher production and percent of daily growth rate in the case of the square net than in the long line method. Our present findings collaborated well with the cultivation of *G.* *dura* in the Gulf of Mannar^21^ and the cultivation of *G.* *edulis* on the Mandapam coast^27^ by the square net method. Once the plants are fully developed, the horizontally positioned square net will provide support while also reducing frond damage and ejecting caused by wave interaction and tidal currents. The expanded surface area also helps to facilitate the formation of new shoots in the case of square net methods. However, because of seedling loss, owing to strong water currents and wave action, the long line approach resulted lesser biomass yielded. A similar lower growth rate was reported in the long line rope method by Paramasivam and Devadoss^28^. In this experiment, we found that both production and %DGR were higher for the square-shaped raft than for the triangular-shaped raft. A contradictory finding has been observed earlier in the case of *G.* *edulis* cultures^29^. This is because we did not perform a cluster management to arrange the triangular rafts. As a result, each triangular raft received individual drag pressures and loss of fragile fronds of seaweed. However, for the cultivation of seaweed, a square-shaped raft was also applied and found to have a satisfactory biomass yield performance, which is correlated to our present findings^22,30^.

Seeding intensity is considered a critical factor for the cultivation of seaweed. The appropriate seeding intensity and seeding distance will enable better water movement that will help to distribute nutrients and advance the propagation, which results in an improvement in growth rates^31^. In our present experiment, we found that 100 seeds (500 g) initial seeding per square meter showed the highest biomass yield performance and %DGR compared to the others. In our study, the cultivation of *G.* *longissima* with an initial 500 g seaweed seed per square meter was supposed to be appropriate. These results mean all the thalli parts received enough sunlight and got enough space to grow. Hence, there is less possibility of self-shading of thalli, which ensures nutrient supply to the individual thalli. There are limited scientific research findings available in this regard in the literature. Though, Mantri et al.^29^ and Islam et al.^17^ used 1 kg fresh weight for seeding in the case of *G.* *edulis* and *Hypnea* sp. cultures which is much higher than our current experiment. These variations might be caused by differences in species, location or culture type. Another factor that has been shown to influence seaweed farming production is the harvesting phase. Harvesting crops on time provides excellent crop quality and profit margin. In this investigation, we observed that harvesting seaweed 30 days after sowing provided the best production output. Seaweed harvested 15 and 45 days after seeding had a lower production efficiency. This might have been caused by the decay of seaweed ropes after 30 days, resulting in a reduction in seaweed production. Our research correlates with the findings of Bokhtiar et al.^19^ in the case of *G.* *tenuistipitata* and Padhi et al.^24^ in the case of *G.* *verrucosa*. Moreover, the harvesting phase is influenced primarily and foremost by the rate of growth and the period when the seaweeds achieve their maximum economic value, such as a high polysaccharide substance or specific flavor qualities^32^. During the 90-day culture period, we found that the seaweed culture at the surface level of the water column had the maximum output and % DGR (12.44 ± 0.48 kg/m^2^, 4.65 ± 0.04% day^−1^), while the depths of 25 cm and 50 cm had much lower output and nil, respectively. At the water surface level, seaweed was highly exposed to sunlight which helped in the photosynthesis process, as well as the comparatively higher water circulation than the other depth carried a higher amount of nutrients assimilated by the seaweed. Additionally, a lower transparency level at the culture site during the experimental period resulted in lower sunlight penetration at 25 cm and 50 cm depth, though other scholars such as Mensi et al.^26^ cultured *G.* *gracilis* at 400 cm depth, Yang et al.^33^ cultured *G.* *lemaneiformis* at 50–150 cm depth, and Ben Said et al.^34^ cultured *G.* *gracilis* at 50 cm depth.

Epiphyte growth has been documented in several other cultivated seaweeds, which are perhaps linked to significant changes in salinity, temperature and nutrient levels^22,35^. In our experiment, the dominance of *Ulva* species was observed throughout the culture period as they are locally available on many shores. They are also stress-tolerant as well as functionally resilient and have been shown to endure a wide range of temperature and irradiance levels^35^. These fouling algae directly compete with *Gracilariopsis* for space in the culture unit. Additionally, the cost–benefit analyses were used to compare the commercial feasibility of these floating raft methods on the coast of Bangladesh. The daily growth rate observed in this floating raft method was about 4.72% day^−1^. The measured daily growth rates during the present study were parallel to those reported by Bermejo et al.^8^ in open coastal waterbody (4.71% day^−1^), He et al.^9^ in an IMTA system with fish species (3.0% day^−1^) and Bermejo et al.^36^ in a nearby traditional salina (3.2% day^−1^). In this study, *G.* *longissima* had long periods of net biomass loss, and net growth rates were not stable over longer periods of time. A similar problem was also stated in the raft culture of *G.* *edulis* in the open sea of India^22^. Using new cuttings at the beginning of each cycle, harvested from young plants that are in the process of actively growing, solves this problem. It was stated that large-scale commercial cultivation of *Gracilaria* is possible when the growth rate was more than 5% per day^37^. Our findings reveal that *G.* *longissima* farming can become a promising sector for large-scale production in our coastal area.

Considering all of these aspects, our research indicated that *G.* *longissima* species may be cultivated using the floating raft method in our coastal region, especially on the Chowfoldondi coast of Cox’s Bazar. However, for better biomass yield performance, certain important aspects such as seeding intensity, raft shape, rope materials, culture type, harvesting phase and water depth need to be considered, as we found that these parameters have a significant influence on the biomass yield performance of *G.* *longissima*. Further research could be performed on the year-round cultivation of seaweed and the biochemical composition, agar content and gel strength of cultured seaweed at altered locations along the Cox’s Bazar coast.

## Methods

### Study area

**Figure 2:**
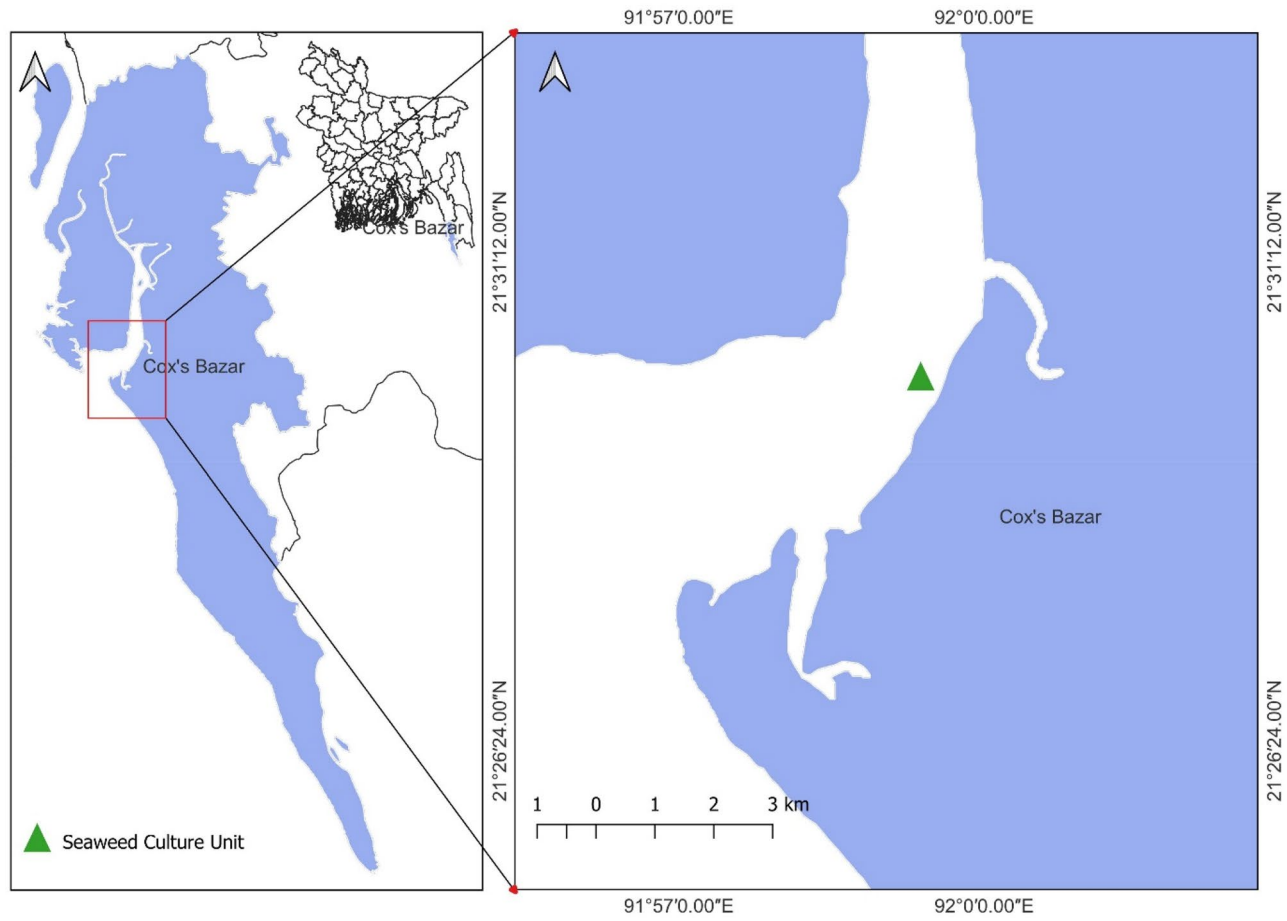
Location of the *G.* *longissima* culture area at Chowfoldondi, Cox’s Bazar (the map was generated by using QGIS version 3.24.1.; retrieved from https://www.filehorse.com/download-qgis/old-versions/).

An experimental seaweed culture site was designed in the sheltered intertidal zones of Chowfoldondi (91°59′38.8′′ E and 21°30′11.7′′ N) of Cox’s Bazar district (Fig. 2), along the Bay of Bengal’s north-eastern coast. The study area was on the Bakkhali River (the second largest river in the district), which has moderate wave action and is directly connected to the Bay of Bengal. The average water depth of the culture site was 700 cm. This area is considered a biologically diverse ecosystem, having intertidal mudflats with various salt marshes, seagrasses, cord grasses, seaweeds and also some mangrove vegetation. Additionally, this place is a safe habitat for numerous types of fish, reptiles, oysters, mussels, crabs, snails, shrimp and so on. The yearly average rainfall and temperature were 3770 mm and 25.6 °C, respectively, in the Cox’s Bazar district. This tropical climatic area’s wind speed average was about 8.3 miles per hour. The study was performed for a period of 90 days from January 2022 to March 2022.

### Seed collection

Young, wild *G.* *longissima* seeds were initially collected from the intertidal zones of the Nuniachara coast (91°57′52.0′′ E and 21°28′28.9′′ N) of Cox’s Bazar Sadar. This sand flat site is a natural bed of seaweed with some other seagrass, salt marsh and mangrove vegetation. Permission to collect seaweed samples and their culture practices was obtained from the local government in accordance with local and national legislation. The botanical identification of seaweed species was checked and confirmed through the published literature^38,39^. Dr. Md. Enamul Hoq, Former Director of BFRI, validated the botanical identification of the seaweed species, as the voucher specimen had already been placed at BFRI herbarium [BFRI (MFTS-RS-18/19-038). A fresh sample was collected in an open box with adequate seawater and an aeration facility and then immediately transferred to the cultivation site to maintain fresh quality.

### Experimental culture raft setup

**Figure 3:**
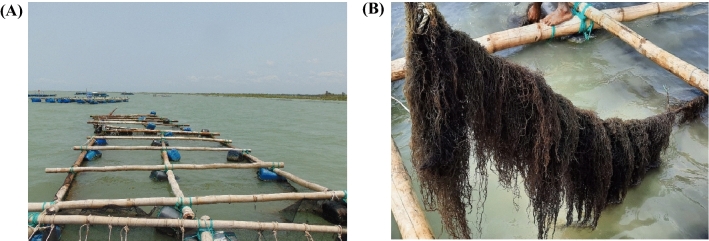
**(A)** Floating raft structure for *G.* *longissima* culture at Chowfoldondi and **(B)** *G.* *longissima* grown in floating raft.

Several bamboo poles (7.0–10.0 cm diameter) made into square (5 m × 5 m) frames were prepared to provide the seaweed culture layout. The four corners of the raft were steadied tightly between and among themselves to keep the raft shape intact. Two more bamboos were fastened tightly at the opposite ends to make the structure stronger. Four recycled plastic drams were attached to the structure's four corners, ensuring that it was always floating on the water. A 1.50 cm mesh size plastic net was placed in the lower part of the frame to minimise the wave action, and crop loss caused by plant rupture from the base, especially during adverse weather. All of the rafts were rope-tied, placed in the culture site and anchored to help stabilise the structure. The anchor of the structure was placed in such a way that it could raise and fall vertically during the tidal action. Each experiment had three replications (Fig. 3).

### Seaweed seeding

The younger pieces of *G.* *longissima* were used for seeding with an average of 5 ± 0.4 g of fresh weight in each knot and 5 cm size in the rope twists. No fertiliser, growth hormone or any other chemicals were used during the culture period. Partial harvesting was done when the seaweed reached an average standard length. The partial harvesting took place by cutting off the algae hanging on the surface, allowing the base on the surface to expand further. Seaweed biomass yield production was measured as the fresh weight of seaweed per unit culture area (kg/m^2^) and was calculated using Eq. (1) ^40^.

1 $${\text{Y }} = \, \left( {{\text{W}}_{{\text{n}}} - {\text{ W}}_{0} } \right)/{\text{A}}$$

Here, Y is the seaweed biomass yield production; W_n_ is the raw weight on day n; W_0_ is the beginning raw weight; A is the culture unit’s area.

Daily growth rate % was calculated using Eq. (2) ^41^.

2 $${\text{DGR }}\% \, = {\text{ ln }}\left( {{\text{W}}_{{\text{f}}} /{\text{ W}}_{{\text{o}}} } \right) \, /{\text{ t }} \times { 1}00$$

Here, W_f_ is the final raw weight (g) at t day; W_o_ is the initial raw weight (g); t is the cultivation period (days).

### Water quality variables

The culture site was studied throughout the 90-day culture period from January 2022 to March 2022. Sampling was carried out twice a month. Multiple water quality parameters such as temperature, pH, dissolved oxygen (DO), salinity, TDS (total dissolved solids), transparency, alkalinity, ammonia, nitrite, nitrate, phosphate and silica were measured. Temperature, pH, dissolved oxygen, salinity and TDS were all measured on the spot using a HANNA HI-98194 multiparameter. Measurement of water transparency was performed with a Secchi disc. Water samples from 0 to 100 cm depth was collected (Van Dorn water sampler) and immediately transported to the laboratory. Alkalinity, ammonia, nitrite, nitrate, phosphate and silica in water were analysed following the methods of HANNA (Hanna COD and Multiparameter Bench Photometer, 230 V-HI83099 procedure manual).

### Effect of rope material

This experiment was carried out to explore the rope material’s effect on the biomass yield performance of *G.* *longissima*. In this study, four types of rope materials (T_1_ = nylon rope, T_2_ = plastic rope, T_3_ = coir rope: acquired from coconut husk, and T_4_ = jute rope) were used. Among them, coir and jute ropes were biodegradable, while the other two were non-biodegradable. The best biomass yield performances along with the rope materials’ sustainability in saline water were observed.

### Effect of culture type

In this study, different culture types, such as long line and square net methods, were used to observe the biomass yield performance of *G.* *longissima*. Here, T_1_ stands for the long line method and T_2_ stands for the square net method.

### Effect of raft shape

In this experiment, different shaped rafts were prepared and observed for their effect on the biomass yield performance of *G.* *longissima*. Here, T_1_ (square shape) and T_2_ (triangular shape) rafts were prepared and observed for their biomass yield performance.

### Effect of seeding intensity

This experiment was conducted to observe the effect of seeding intensity on the biomass yield production of *G.* *longissima*. Here, T_1_, T_2_ and T_3_ indicate 50, 100 and 150 seeds per square meter, respectively. This results in 250 g, 500 g and 750 g initial seaweed seeds per square meter for cultivation. During harvesting, fresh seaweed samples were collected separately and washed carefully with running fresh water to remove dirt and any other impurities. The fresh sample was then weighted with the help of a digital balance.

### Effect of harvesting phase

In this study, after the initial seeding, harvesting was performed at four different intervals (T_1_ = 15 days, T_2_ = 30 days, T_3_ = 45 days and T_4_ = 90 days). Here, in the case of T_1_, a total of six partial harvests; in the case of T_2_, a total of three partial harvests; in the case of T_3_, a total of two partial harvests; and in the case of T_4_, zero partial harvests were performed throughout the 90 day of the culture period. The best harvesting intervals were evaluated through the maximum biomass yield performance of *G.* *longissima*.

### Effect of water depth

In this study, several vertical culture units were placed at different water levels in the open sea. T_1_ represents the first unit that was placed at the water's surface, T_2_ represents the second unit that was placed below the first one at a 25 cm depth from the water's surface, and T_3_ represents the third unit that was placed below the second one at a 50 cm depth from the water's surface. The possibility of vertical expansion of seaweed culture was evaluated through the maximum biomass yield performance of *G.* *longissima* at each depth.

### Seasonal appearance of epiphytic algae

Epiphytic algal occurrences on *G.* *longissima* and the associated culture materials were documented during the 90-day (January–March 2022) cultivation period. Seaweed samples were collected, washed with clean water and preserved in silica gel in sample vials. Finally, seaweed samples were confirmed through microscopic examination and cross-checked against the published literature^38,39^.

### Cost–benefit analysis of seaweed culture

The cost–benefit analyses of *G.* *longissima* cultures for 20 rafts (5 m × 5 m) in a 180-day culture period was examined. The economic turnover of *G.* *longissima* cultures through biomass yield production can be estimated. Investment includes all input materials and maintenance costs. Based on the local market, all prices of investments were included and expressed in US$.

### Statistical analysis

The experimental data was analysed using standard statistical techniques. Statistical package SPSS version 20.0 (IBM Co., Chicago, IL) was used to examine the data. One way ANOVA and T-test were used to determine the significance of each parameter among different treatments. The level of significance was set at a 95% probability level.

## Data Availability

All data generated or analyzed during this study are included in this published article.

## References

[1] Pereira L, Gheda SF, Ribeiro-Claro PJ (2013). Analysis by vibrational spectroscopy of seaweed polysaccharides with potential use in food, pharmaceutical, and cosmetic industries. Int. J. Carbohydr. Chem..

[2] Sobuj MKA (2021). Evaluation of bioactive chemical composition, phenolic, and antioxidant profiling of different crude extracts of *Sargassum*
*coriifolium* and *Hypnea*
*pannosa* seaweeds. J. Food Meas. Charact..

[3] Sobuj MKA (2021). Effect of solvents on bioactive compounds and antioxidant activity of *Padina*
*tetrastromatica* and *Gracilaria*
*tenuistipitata* seaweeds collected from Bangladesh. Sci. Rep..

[4] Mollet JC, Rahaoui A, Lemoine Y (1998). Yield, chemical composition and gel strength of agarocolloids of *Gracilaria*
*gracilis*, *Gracilariopsis*
*longissima* and the newly reported *Gracilaria* cf. *vermiculophylla* from Roscoff (Brittany, France). J. Appl. Phycol..

[5] Cabral P, Levrel H, Viard F, Frangoudes K, Girard S, Scemama P (2016). Ecosystem services assessment and compensation costs for installing seaweed farms. Mar. Policy..

[6] FAO. *The**State**of**World**Fisheries**and**Aquaculture**2020.**Sustainability**in**Action.**Rome*. 10.4060/ca9229en (2020).

[7] Pereira, R. & Yarish, C. Mass production of marine macroalgae. in *Encyclopedia**of**Ecology*. 2236–2247. 10.1016/B978-008045405-4.00066-5 (Elsevier, 2008).

[8] Bermejo R, Cara CL, Macías M, Sánchez-García J, Hernández I (2020). Growth rates of *Gracilariopsis*
*longissima*, *Gracilaria*
*bursa-pastoris* and *Chondracanthus*
*teedei* (Rhodophyta) cultured in ropes: implication for N biomitigation in Cadiz Bay (Southern Spain). J. Appl. Phycol..

[9] He Q, Zhang J, Chai Z, Wu H, Wen S, He P (2014). *Gracilariopsis*
*longissima* as biofilter for an integrated multi-trophic aquaculture (IMTA) system with *Sciaenops*
*ocellatus*: Bioremediation efficiency and production in a recirculating system. Indian J. Mar. Sci..

[10] Hernández I, Fernández-Engo MA, Pérez-Lloréns JL, Vergara JJ (2005). Integrated outdoor culture of two estuarine macroalgae as biofilters for dissolved nutrients from *Sparus*
*auratus* waste waters. J. Appl. Phycol..

[11] Bezerra AF, Marinho-Soriano E (2010). Cultivation of the red seaweed *Gracilaria*
*birdiae* (Gracilariales, Rhodophyta) in tropical waters of northeast Brazil. Biomass Bioenergy.

[12] Rejeki S, Ariyati RW, Widowati LL, Bosma RH (2018). The effect of three cultivation methods and two seedling types on growth, agar content and gel strength of *Gracilaria*
*verrucosa*. Egypt. J. Aquat. Res..

[13] Marinho-Soriano E, Moreira WSC, Carneiro MAA (2006). Some aspects of the growth of *Gracilaria*
*birdiae* (Gracilariales, Rhodophyta) in an estuary in Northeast Brazil. Aquac. Int..

[14] Kaliaperumal N, Rajagopalan MS, Chennubhotla VSK (1992). Field cultivation of *Gracilaria*
*edulis* (Gmelin) Silva in Minicoy lagoon (Lakshadweep). Seaweed Res. Util..

[15] Kim JH, Lee SD, Choi S, Chung IK, Shin J (2005). Cultivation of *Gracilaria*
*chorda* (Gracilariales, Rhodophyta) by vegetative regeneration. Algae.

[16] Veeragurunathan V, Prasad K, Malar Vizhi J, Singh N, Meena R, Mantri VA (2019). *Gracilaria*
*debilis* cultivation, agar characterization and economics: Bringing new species in the ambit of commercial farming in India. J. Appl. Phycol..

[17] Islam MM, Khan MSK, Hasan J, Mallick D, Hoq ME (2017). Seaweed *Hypnea* sp. culture in Cox’s Bazar coast, Bangladesh. Bangladesh J. Zool..

[18] Hoq ME, Haque MA, Islam MM (2016). Feasibility of seaweed culture in Inani and Bakkhali Coast of Cox’s Bazar, Bangladesh. Pak. J. Mar. Sci..

[19] Bokhtiar SM (2022). Yield improvement of *Gracilaria*
*tenuistipitata* by optimizing different aspects in coast of Cox’s Bazar, Bangladesh. Sci. Rep..

[20] Lee WK, Lim PE, Phang SM, Namasivayam P, Ho CL (2016). Agar properties of *Gracilaria* species (Gracilariaceae, Rhodophyta) collected from different natural habitats in Malaysia. Region. Stud. Mar. Sci..

[21] Veeragurunathan V (2015). Feasibility of *Gracilaria*
*dura* cultivation in the open sea on the Southeastern coast of India. Aquaculture.

[22] Ganesan M, Sahu N, Eswaran K (2011). Raft culture of *Gracilaria*
*edulis* in open sea along the south-eastern coast of India. Aquaculture.

[23] Veeragurunathan V, Prasad K, Singh N, Malarvizhi J, Mandal SK, Mantri VA (2016). Growth and biochemical characterization of green and red strains of the tropical agarophytes *Gracilaria*
*debilis* and *Gracilaria*
*edulis* (Gracilariaceae, Rhodophyta). J. Appl. Phycol..

[24] Padhi S (2011). Cultivation of *Gracilaria*
*verrucosa* (Huds) Papenfuss in Chilika Lake for livelihood generation in coastal areas of Orissa State. J. Appl. Phycol..

[25] Freitas, M. A. D. S. V. S. D. The biotechnological potential of the seaweed *Gracilariopsis**longissima* (Rhodophyta, Gracilariales). in *Doctoral**Dissertation* (2017).

[26] Mensi F, Nasraoui S, Bouguerra S, Ben Ghedifa A, Chalghaf M (2020). Effect of lagoon and sea water depth on *Gracilaria*
*gracilis* growth and biochemical composition in the northeast of Tunisia. Sci. Rep..

[27] Rao MU (1974). On the cultivation of *Gracilaria* in the near shore around Mandapam. Curr. Sci..

[28] Paramasivam M, Devadoss CGM (1987). Field cultivation of *Gracilaria*
*edulis* (Gmelin) Silva in Chinnapalam estuary, Pamban. J. Mar. Biol. Assoc. Ind..

[29] Mantri VA, Ashok KS, Saminathan KR, Rajasankar J, Harikrishna P (2015). Concept of triangular raft design: Achieving higher yield in *Gracilaria*
*edulis*. Aquac. Eng..

[30] Thirumaran G, Anantharaman P (2009). Daily growth rate of field farming seaweed *Kappaphycus*
*alvarezii* (Doty) Doty ex P. Silva in Vellar Estuary. World J. Fish Mar. Sci..

[31] Neish, I. C. The *Eucheuma**Seaplant**Handbook*. Vol. I. *Agronomy,**Biology**and**Culture**System.**Seaplantnet**Technical**Monograph*. 1–36 (2005).

[32] Titlyanov EA, Titlyanova TV (2010). Seaweed cultivation: Methods and problems. Russ. J. Mar. Biol..

[33] Yang YF (2006). Growth of *Gracilaria*
*lemaneiformis* under different cultivation conditions and its effects on nutrient removal in Chinese coastal waters. Aquaculture.

[34] Ben Said R (2018). Effects of depth and initial fragment weights of *Gracilaria*
*gracilis* on the growth, agar yield, quality, and biochemical composition. J. Appl. Phycol..

[35] Fletcher RL (1995). Epiphytism and fouling in *Gracilaria* cultivation: An overview. J. Appl. Phycol..

[36] Bermejo R, Macías M, Cara CL, Sánchez-García J, Hernández I (2019). Culture of *Chondracanthus*
*teedei* and *Gracilariopsis*
*longissima* in a traditional salina from southern Spain. J. Appl. Phycol..

[37] Anderson RJ, Levitt GJ, Share A (1996). Experimental investigations for the mariculture of *Gracilaria* in Saldhana Bay, South Africa. J. Appl. Phycol..

[38] Sarkar MSI, Kamal M, Hasan MM, Hossain MI (2016). Present status of naturally occurring seaweed flora and their utilization in Bangladesh. Res. Agric. Livest. Fish..

[39] Islam MM (2019). Seaweeds of Bangladesh Coast.

[40] Doty MS (1986). Estimating returns from producing *Gracilaria* and *Eucheuma* on line farms. Monogr. Biol..

[41] Dawes CJ, Kovach C, Friedlander M (1993). Exposure of *Gracilaria* to various environmental conditions. 2. The effect on fatty acid composition. Bor. Mar..

